# Comparative evaluation of the effect of impression materials and trays on the accuracy of angulated implants impressions 

**DOI:** 10.4317/jced.55227

**Published:** 2018-11-01

**Authors:** Hakimeh Siadat, Zeinab Saeidi, Marzieh Alikhasi, Somayeh Zeighami

**Affiliations:** 1 DDS, MSc; Full Professor, Dental Implant Research Center Dentistry Research Institute and Department of Prosthodontics, School of Dentistry, Tehran University of Medical Sciences, Tehran, Iran; 2 DDS, MSc; Assistant Professor, Department of Prosthodontics, School of Dentistry, Kerman University of Medical Sciences, Kerman, Iran; 3 DDS, MSc; Associate Professor, Dental Implant Research Center Dentistry Research Institute and Department of Prosthodontics, School of Dentistry, Tehran University of Medical Sciences, Tehran, Iran; 4 DDS, MSc; Associate Professor, Dental Implant Research Center Dentistry Research Institute and Department of Prosthodontics, School of Dentistry, Tehran University of Medical Sciences, Tehran, Iran

## Abstract

**Background:**

Vinyl Polyether Siloxane is a newly introduced impression material and studies on that is scarce. Implant insertion in posterior mandible might be angulated due to anatomical considerations. The purpose of this study was to compare the dimensional and angular accuracy of impressions using full-arch versus sectional tray and Vinyl Polysiloxane versus Vinyl Polyether Siloxane in angulated implants.

**Material and Methods:**

Four implants were placed in dental areas #19, #21, #28 and #30 of a Kennedy class I mandibular acrylic model with 30° lingual angulation. Twenty sectional and 20 full-arch open trays were made on the primary cast. Impressions were taken using Vinyl Polysiloxane and Vinyl Polyether Siloxane (n=10 in 4 groups); and were poured with type IV dental stone. The coordinate measuring machine was used to measure displacements in the X, Y and Z axes and rotational discrepancies of implants. The data were analyzed using SPSS 22 and two-way ANOVA.

**Results:**

Type of tray had no significant effect on the dimensional and angular accuracy of impressions (*p* >0.05). Type of impression material significantly affected linear displacement (∆r) (*P* <0.05); but it did not significantly affect the rotational displacement (*P* >0.05).

**Conclusions:**

Vinyl Polysiloxane yielded more accurate impressions of angulated implants.

** Key words:**Dental implant, impression material, impression tray, vinyl polysiloxane, vinyl polyether silicone, coordinate measuring machine.

## Introduction

Dental implant in the alveolar bone is not surrounded by the periodontal ligament, and assembly of prosthesis over the implant yields a structure composed of prosthetic superstructure, implant fixture and bone as one unit (1). Prosthesis-implant misfit causes internal stresses in these three components (1). Stresses due to the lack of passivity of prosthesis lead to mechanical and biological complications (2). Accurate impression taking and transfer of implant position to the master cast is an important step in fabrication of prosthesis and achieving optimal fit. Impression technique and material are two important factors in obtaining precise fit of implant prostheses (3,4). In general, two techniques are used for implant impression taking (direct or open tray and indirect or closed tray); Direct technique could be done with or without splinting (5). In previous studies the accuracy of impressions taken from angulated implants were same in direct and indirect techniques (5). Furthermore, there is no consensus about splinting in angulated implants before impression taking (5).

Metal and plastic stock trays and custom trays have also been compared for implant impression taking (6). To minimize impression errors, most studies recommend using a rigid tray irrespective of the type of impression material (6). On the other hand, special acrylic trays have been produced more accurate casts in comparison to the stock trays; because of rigidity and uniform thickness of impression material (6). However, studies on the use of sectional trays are scarce. Geramipanah *et al.* compared two types of trays and found no significant difference in the accuracy of impressions taken with sectional and full arch trays (2). It is assumed that during taking impressions from angulated implants, full-arch trays would undergo greater distortion when removing the tray from bilateral undercuts and consequently, greater stress is created in the impression material, which may cause inaccuracies. In such cases, use of sectional tray may decrease impression errors since it covers fewer implants and is also less flexible than the full-arch tray (2).

Type of impression material is another important factor affecting the accuracy of impressions. In general, polyether and Vinyl Polysiloxane (VPS) are considered as the choice impression materials (6). Polyether present dimensional stability and tear resistance and also rigidity; while, VPS has better hydrophilicity and higher dimensional stability (6). In a systematic review, Lee *et al.* did not find any significant difference in the accuracy of these impression materials (3). Due to optimal elastic recovery, VPS is considered as a preferred impression material for taking impressions from angulated implants (4,7,8). Recently, Vinyl Polyether Siloxane (VPES) was introduced to the market, which is a combination of VPS and polyether and has some of the properties of both materials. The manufacturer claims that this material has very good flowability and hydrophilicity. Also, this material has double snap property, which decreases the distortion of impression upon completion of working time due to changed viscosity and subsequent formation of cross-links. Also, this material has optimal elasticity and is tasteless and odorless.

Considering the information gap on the dimensional and angular accuracy of impressions taken with VPES and sectional tray, this study aimed to assess the accuracy of impressions taken from bilateral angulated implants using sectional versus full arch trays and PVES versus VPS impression materials. The null hypothesis was that type of tray and impression material would have no effect on the dimensional and angular accuracy of implant impressions.

## Material and Methods

**Figure 1:**
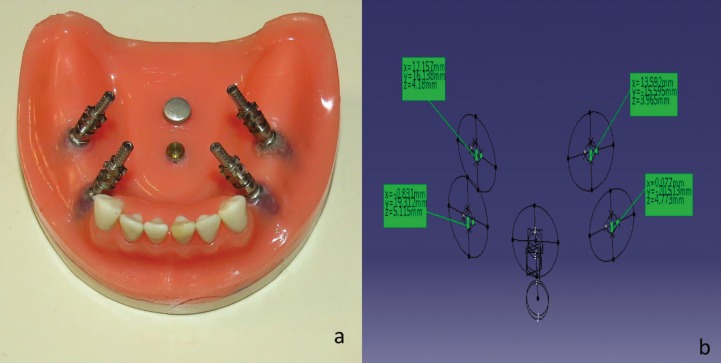
(a) Master model with four angled implants (b) Measurement of displacements in x, y, and z directions and rotational displacement by CMM.

A Kennedy class I mandibular acrylic model (Parsateb, Tabriz, Iran) was used in this *in vitro* experimental study. In order to determine the position of implants, teeth arrangement on acrylic base was done and left and right mandibular first premolar and molar teeth (dental areas #19, #21, #28 and #30) were selected as implant insertion areas. Then on each side of edentulous ridge 2 parallel (mesiodistal angulation of implant is not common due to anatomical limitations) holes with 30° lingual angulation were prepared (this is the worse case of angulated implants in posterior mandible). Four implants (Implantium, Dentium, Seoul, South Korea) measuring 4.3 mm in diameter and 12 mm in height were inserted at the level of ridge using self cure acrylic resin (Technovit 4000; Heraeus Kulzer GmbH & Co., Wehrheim, Germany). Two metal rods were placed at the center of the original model and planes trace by these rods served as the reference planes for measurements in X, Y and Z axes. The reference point for measurement was the center of anterior rod. Since the position of these cylinders transfer by the impression, we will be able to calculate the coordinates of each implant in each sample irrespective of the 3D displacement of the other implants. As baseline data, rotational (θ) and dimensional (X, Y and Z) coordinates of implants were measured using coordinate measuring machine (CMM) (Global DEA, Hexagon, Italy) with 4μm accuracy and a SP25 probe with 1mm diameter (Fig. 1a,b). The machine was first calibrated by placing the probe tip on the calibration ball.

One of the most accurate coordinating tools is the Coordinate Measuring Machine. CMM measure physical and geometrical properties of the sample. The measurements are performed by touching a point using a probe and displaying the coordinates of each point in X, Y, Z axes.

To measure the sample’s coordinates, at first the reference planes (X, Y, and Z) were defined. For this purpose, the cylinders located at the center of the model were scanned by the machine. The Z plane was defined on the surface of the anterior cylinder. X plane was defined as a line passing through two cylinders and perpendicular to the Z plane. The Y plan was perpendicular to the other two planes. Impression copings were closed on the implants and each implant was scanned circularly and the center of implants was used to determine the X, Y and Z coordinates.

To measure the angular coordinate of the implants, a delicate flat blade was made. Implants have a hexagon connection. The edge of blade was placed at an angle between the two sides of the hexagon and the other edge of the blade was used to measure its angle with the X axis.

For the same seating of the spatial trays, four stops were made in anterior, posterior and lateral sides of master model using autopolymerizing acrylic resin.

**Figure 2:**
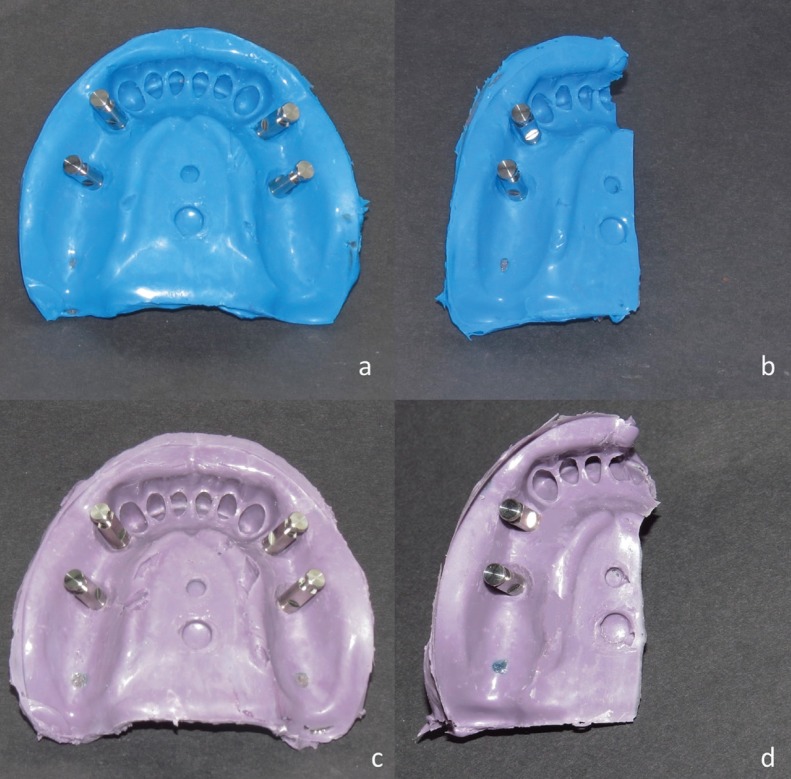
Impressions made by (a) full-arch tray and PVS material, (b) sectional tray and PVS material, (c) full-arch tray and PVES material, (d) sectional tray and PVES material.

Four transfer impression copings (DTF 45 11 HL, Implantium, Dentium, Seoul, South Korea) were tightened on implants and a closed tray impression was taken from the model by a stock tray and irreversible hydrocolloid (Alginoplast, Heraeus Kulzer GmbH & Co., Wehrheim, Germany). Primary cast was poured with dental stone type III (Tara 250, Kheyzaran, Isfahan, Iran). Two layers of base plate wax (Dentsply, Weybridge, United Kingdom) were adapted on the cast and in order to keep the material thickness at 3 mm, three tissue stops were prepared (one on incisal edge and two on posterior ridge). light-polymerized resin (Megatray, Megadenta, Redeberg, Germany) was used and 20 full-arch and 20 sectional open trays were made with a thickness of 2mm. The sectional tray was fabricated such a way to cover the two implants placed in the right quadrant. Thirty minutes prior to taking the impression, the internal surface and 5mm border of the external surface of each tray were covered with silicone adhesive (Kettenbach GmbH & Co. KG, Eschenburg, Germany). Pick-up impression copings (DPU 45 11 HL, Implantium, Dentium, Seuol, South Korea) were screwed onto the implants with 10 Ncm torque (2). Impressions were taken by a single operator and using regular consistency of VPS (Panasil, Kettenbach GmbH & -Co. KG, Eschenburg, Germany) (Fig. 2a,b) and medium consistency of VPES (EXA’lence, GC Corporation, Tokyo, Japan) (Fig. 2c,d). Impression gun was used for mixing. Impressions were taken at 23±1°C temperature and 50±10° relative humidity (9). To apply standard load, 1.5kg weight was placed on each tray during the process of polymerization (2). The impression-tray was immersed in water at 36±1°C during the process (2). The path of insertion and removal of trays were in vertical direction.

**Figure 3:**
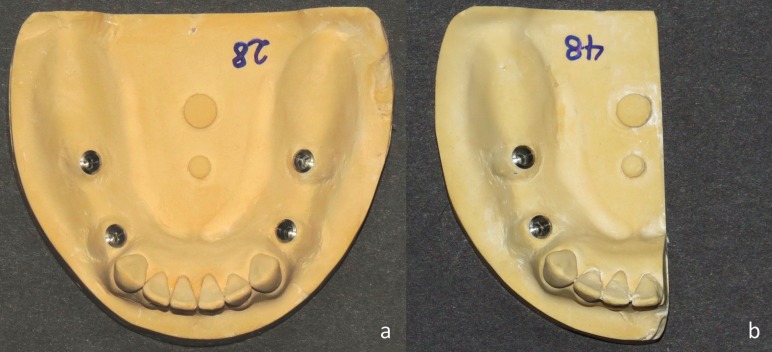
(a) Final full-arch cast. (b) Final sectional cast.

In total, 40 impressions were taken in 4 groups (n=10 in each group) using two types of trays and two types of impression materials. Implant analogs were connected to impression copings in the impressions and then, beading and boxing were done for each impression. After one hour, the impressions were poured with type IV dental stone (Fujirock EP, GC Corporation, Tokyo, Japan). After 40 minutes (as recommended by the manufacturer), the casts were separated from the impressions and coded (Fig. 3a,b).

**Figure 4:**
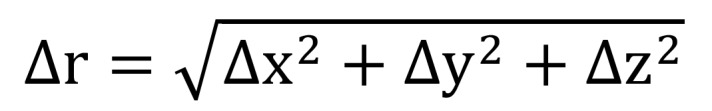
Formula.

The coordinates of each analog were measured using a CMM (Global DEA, Hexagon, Italy). The distance from the center of each analog to the reference point (center of anterior cylinder) in the X, Y and Z axes and also their angular coordinates (θ) were measured. The measurements were repeated on each analogue three times, and the mean of each of the measurements in each analogue was compared with those obtained from the reference model. To calculate 3D displacement, the following equation was used: (Fig. 4):

Due to normal distribution, the data were analyzed using SPSS version 22 (SPSS Inc., IL, USA) and two-way ANOVA test considering the type of tray and type of impression material. Level of significance was 0.05.

## Results

Mean and standard deviation of displacements in X, Y, Z axes, linear (∆r) and rotational discrepancies (∆θ) have been summarized in  Table 1. *P*-values of each variable are shown in  Table 2.

**Table 1:**
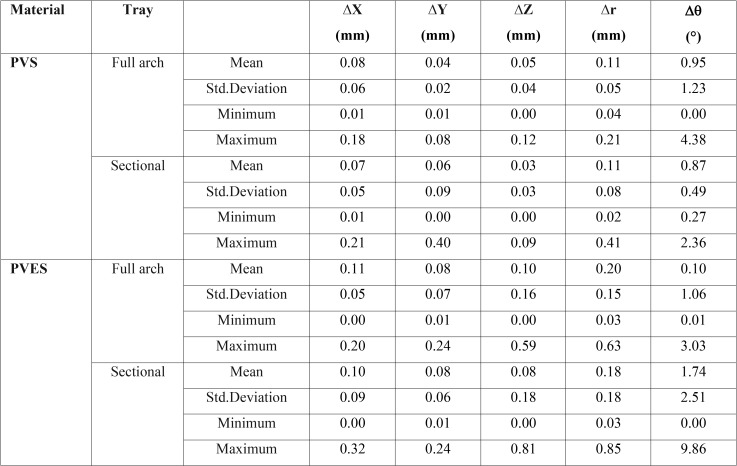
Mean and standard deviation of dimensional (∆X, ∆Y, ∆Z), linear (∆r) and angular (∆θ) displacements for trays and materials.

**Table 2:**
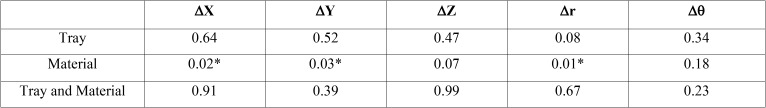
*P*-values of displacements in X, Y, Z axes and linear (∆r) and angular discrepancies (∆θ) for tray, material and their interaction.

The results showed that there were no statistically significant differences in dimensional (X, Y and Z axes), linear (∆r) and rotational discrepancies (∆θ) between full-arch and sectional trays (*p*>0.05).

Type of impression material significantly affected the dimensional accuracy of impressions in X, Y axes and linear displacement (∆r) (*p*<0.05), but it had no significant effect on displacement in Z axis and angular discrepancy (∆θ) (*p*>0.05). Also, the interactions between tray and material had no significant effect on dimensional and angular accuracy of impressions (*p*>0.05).

## Discussion

Taking accurate impression is the first step in fabrication of a high-quality prosthetic restoration with precise fit. Impression material and technique play a fundamental role in obtaining accurate impressions (10). This study evaluated the accuracy of impressions taken with two types of trays (full-arch and sectional) and two types of impression materials (VPS and VPES).

In the posterior mandible, lingual undercuts are present due to the position of submandibular glands. In order to prevent perforation of the lingual plate, implants are placed angulated in thids region (11). Morphological assessment of mandibular ridges in a previous study revealed that posterior mandibular undercuts were present in 66% of the patients above the inferior alveolar canal and the mean angulation in this area was 57° (11). After tooth extraction, mandibular ridge undergoes resorption of the crestal bone and lingual plate, and depth of undercuts decreases. Implants are often placed in more than 25° angulations in the clinical setting (2). In the current study, implants were placed with 30° lingual angulation to simulate angular placement of implants due to the presence of undercuts in the posterior mandible.

The results of this study showed that impressions taken with full-arch and sectional trays had no significant difference in dimensional (X, Y and Z axes), linear (∆r) and angular discrepancies (∆θ). These findings confirmed the null hypothesis of the study stating that the accuracy of impressions would not be affected by the type of tray. Germipanah *et al.* evaluated the accuracy of impressions taken from angulated implants (Replace, Nobel Biocare) using sectional and full-arch trays and found no significant difference in terms of dimensional or angular discrepancies (2). This result may be due to the short connection length of impression copings of the implant system, which enabled easy removal of the impression despite the presence of angulated implants; as a result, the stresses between the coping and impression material were minimized upon removal of the full-arch tray from angulated implants. In their study, the length of connection of copings (Replace system) was 1mm; this value was 1.2mm in the current study using the Implantium system. Alikhasi *et al.* evaluated the effect of 1, 1.5 and 2mm connection lengths of open impression copings on the accuracy of impressions taken from angulated implants and reported no significant difference in the accuracy of impressions when impression copings with less than 2mm connection length were used (12). However, use of copings with longer connections or higher number of implants in future studies may yield significant differences.

The impressions taken with VPS and VPES impression materials yielded significantly different dimensional accuracy in X, Y axes and linear displacement (∆r) (*P*<0.05); however, the difference in Z axis and rotational displacement (∆θ) was not significant. As seen in table 1, the dimensional and angular accuracy of VPS were higher than VPES. Based on these findings, the null hypothesis, stating that the two impression materials would have similar accuracy, was refuted.

Kurtulmus *et al.* compared the accuracy of implant impressions taken with VPS, VPES and polyether and reported that for angulated implants, VPS impressions had higher accuracy than the other two. This has been justified by higher elastic recovery of VPS (7). Vojdani *et al.* assessed the accuracy of impressions taken from straight and angulated implants using VPES, VPS and polyether and showed in angulated implants, VPS yielded the highest accuracy followed by VPES and polyether, respectively (13). A possible explanation would be that elastic recovery is an important property of impression materials, which maintains the dimensional stability of impression when removing the tray and avoids distortion that may occur in removal of impression material due to the angulation of implants (12,14). This distortion has a direct correlation with the number of implants and their angulation (12). Using an impression material with such properties decreases permanent distortion due to less stress between the impression coping and the material, and the accuracy of impressions increases as such (4,12).

The interactions between tray and impression material had no significant effect on dimensional and angular accuracy of impressions. It means that considering both factor (tray and material), the accuracy of impressions was not significantly different.

*In vitro* design was the main limitations of this study. Impressions are taken ideally *in vitro* due to the absence of soft tissue, blood and saliva while these factors in the clinical setting can significantly affect the accuracy of impressions.

There is no agreement about acceptable level of implant framework misfit. In 1983, Bernanke was the first person who quantify the passive fit, and stated that misfit less than 10µm is acceptable. While in 1991, Jemt stated that misfit less than 150 µm is acceptable (15). The framework fitness is affected by factors such as the number and distribution of implants, the rigidity of the framework, the margin location, and the ability of the screw to close the gap (16). The evaluation of the framework fitness in clinic could be done in several ways, like as: alternative finger pressure, direct vision, tactile sensation, radiography, Sheffield test, screw resistance test, disclosing media and 3-Dimensional quantifying systems (15).

It is quoted that unavoidable displacement in the implant impression is about 50 µm (15), But there is no enough information about that amount of analogues displacement after impression making in current study will cause how much misfit in frameworks. It needs to make framework on the final casts and then evaluate the misfit by methods that mentioned above. It will be possible in future studies.

Also, further studies on the accuracy of VPES and sectional trays are required. In addition, future studies must focus on different shapes of dental arches, higher number of implants, variable angulations of implants and different implant systems to better elucidate this topic.

The clinical implication of this study is that since the accuracy of full arch and sectional trays was same, and given that by full arch cast making record base for recording is easier, for impression making of angulated implants in class I Kennedy, full arch tray and VPS is recommended.

## Conclusions

Considering the limitations of this *in vitro* study, following conclusions were obtained.

Type of tray had no significant effect on linear displacement (∆r).

Type of impression material significantly affected linear displacement (∆r).

Rotational discrepancy (∆θ) was not affected by type of tray and material.

VPS was more accurate impression material in angulated implants compared to VPES.
